# Feasibility and acceptability of a life skills and reproductive health empowerment interventionfor young newly married women in Rajasthan, India: A pre-post convergent mixed methods pilot study

**DOI:** 10.21203/rs.3.rs-4255712/v1

**Published:** 2024-10-16

**Authors:** Lakshmi Gopalakrishnan, Sumeet Patil, Debangana Das, Anshuman Paul, Payal Sharma, Ankur Kachhwaha, Usha Choudhary, Nadia Diamond-Smith

**Affiliations:** University of California San Francisco; Network for Economics Engineering Research and Management: Neerman; Network for Economics Engineering Research and Management: Neerman; Network for Economics Engineering Research and Management: Neerman; Orange Tree Foundation; Orange Tree Foundation; Vikalp Sansthan; University of California San Francisco

**Keywords:** ntraception, family planning, adolescent girls, married girls, empowerment interventions, gender equality, health education, rural and tribal communities, South Asia

## Abstract

**Background:**

Newly married young women face increased susceptibility to adverse health outcomes, social isolation, and disempowerment, yet interventions targeting this vulnerable group remain limited. We examined the feasibility and acceptability of TARANG, a life skills and reproductive health empowerment intervention, developed for and with young newly married women.

**Methods:**

We recruited 42 newly married women as participants in our study. We conducted a convergent mixed-methods, single-group cluster pilot study to the intervention in which 41 participants (retention rate=97.6%) completed both baseline and endline surveys in July 2023 and January 2024, respectively. We assessed three primary outcomes––feasibility using proportion of participants who completed at least 50% sessions, acceptability using proportion completely satisfied/somewhat satisfied with TARANG and usefulness using proportion who found TARANG useful/somewhat useful. We conducted in-depth interviews with a purposive sample of 12 participants to understand intervention acceptance and usefulness of the intervention and 6 program staff and moderators to understand barriers to implementing the intervention. We analyzed the quantitative data with descriptive statistics and qualitative data using thematic analysis. We triangulated data from monitoring data filled by moderators, quantitative surveys with participants, and qualitative data.

**Results:**

In the pilot study, 35/42 participants had completed at least one session. Overall, 82% participants attended at least 50% of the sessions. Among those who attended at least 1 session,97% were satisfied/somewhat satisfied with the intervention and 100% of participants found the intervention to be useful/somewhat useful. Qualitative findings reveal participants’ appreciation for open discussions on sensitive topics, such as family planning, and highlight the intervention’s role in filling knowledge gaps and fostering social connections, better sense of agency and improved relationships with mothers-in-law and husbands. While barriers to consistent participation were identified, feedback informed refinements to the intervention for the main trial, including session modifications, increased engagement strategies, and integration of educational videos.

**Conclusion:**

Our community-based participatory approach, developed with input from end users, demonstrated not only high acceptability and feasibility but also had many benefits for newly married women in rural/tribal Rajasthan. Our findings also led to adaptations that may enhance delivery of, and satisfaction with TARANG intervention, which will be tested with a larger sample in a rigorous cluster randomized controlled trial in Rajasthan, India.

**ClinicalTrials.gov:**

The study is registered at ClinicalTrials.gov (NCT06320964). Registered retrospectively on 13 March 2024, https://clinicaltrials.gov/study/NCT06320964.

## 1 Introduction

Marriage is near-universal in South Asia, including India, with a quarter of women aged 20–24 married before the legal age of 18 years.[1] Marriages are “arranged” in this setting, where parents decide on both the timing of marriage and the choice of spouse with minimal input from young women themselves [2, 3] Patrilocality, a common practice in India, further compounds challenges for newlywed brides, who relocate to their husband’s household to reside with in-laws and experience lower empowerment in terms of their decision-making power, freedom of movement, and autonomy. [4, 5] These restrictions imposed on young married women not only limit their ability to act on health knowledge but also hinder opportunities to discuss fertility desires and family planning methods. [6, 7]

Societal pressures to prove fertility early contribute to a high prevalence of childbearing immediately after marriage, with 24% of women under 25 giving birth by age 20. [1, 8] Nearly two-thirds of young married women (15–24 years) in rural areas reported not using any contraceptive method, with 17% of users adopting traditional contraceptive methods.[1] Many barriers prevent uptake of contraceptives among married women, including pro-natal social norms, fear of being labeled “infertile”, the desire for sons, poor spousal communication on family planning, fear of side effects from reversible methods, and low women’s autonomy over reproductive health and decision-making. [9–12] Indeed, norms against the use of family planning and pro-natal norms are so powerful that they prevent local community health workers from counseling newly married women about family planning before their first pregnancy.[11] Such deeply rooted norms around proving fertility immediately after marriage often lead to young married women having children earlier than they would have wanted, with negative effects for both the mother and the child. [13] In addition, many young married women are isolated in their marital homes with low social and peer support and reduced connections with their natal kin, adversely impacting family planning use. [7, 14] Despite the vulnerabilities faced by married adolescent girls and young women, interventions in India have primarily targeted at unmarried adolescents’ empowerment, gender attitudes, and aspirations. [15–17] Without targeted interventions to address the needs of already married adolescent girls and young women, who are particularly vulnerable to unintended pregnancy, social isolation, and disempowerment, we lack the necessary empirical evidence to effectively support this neglected demographic.

Community-based interventions involving women’s groups to improve women’s health and empowerment have a longstanding history in India with two Indian flagship programs, including the National Health Mission and National Rural Livelihoods Mission. Participatory learning and action groups led by Accredited Social Health Activists (ASHAs) under the National Health Mission have demonstrated improved maternal and newborn health in rural eastern India. [18–21] Concurrently, the National Rural Livelihoods Mission has fostered the formation of over 9 million women’s self-help groups focusing on collective savings, credit, and livelihood activities to empower women individually and collectively.[22] Few studies have explored the effectiveness of layering health and nutrition interventions onto SHGs, reporting improvements in contraceptive use and maternal and child health outcomes, alongside cost-effectiveness. [23–25] Further, a systematic review on Indian community-based women’s group interventions identified 19 randomized controlled trials providing evidence of improved perinatal practices, neonatal survival, immunization rates, and dietary diversity among women and children, while highlighting the lack of impact on changing gender norms and addressing violence against women. [26] Nonetheless, none of these studies specifically targeted the needs of young, newly married adolescent girls and young women, echoing the Lancet Commission’s observation on the scarcity of published literature on effective interventions for adolescents and young adults. [27]

Adolescents and young adults (10–24 years), undergo profound physical, cognitive, emotional, and social changes, making them particularly receptive to learning life skills into their daily lives. [27] Broadly defined, life skills refer to adaptive and positive behaviors that empower individuals to effectively navigate the demands and challenges of everyday life. [28] Life skills education trains individuals about risk-taking behaviors and helps them develop communication, assertiveness, self-awareness, interpersonal relationship skills, decision-making, problem-solving, critical thinking, and creative thinking to protect themselves from abuse and exploitation. [29, 30] There is a growing body of evidence that suggests that life skills education can play an important role in empowering girls and women to make informed decisions about their sexual reproductive health and change gender attitudes [31, 32] as well as improve mental health outcomes such as anxiety and depression among in-school adolescents [33]. Despite the evidence that life skills education have major health benefits, previous attempts at life skills interventions from developing countries have been directed at unmarried adolescents enrolled in school settings, overlooking the critical transition period of marriage. [30] We are aware of one life skills-based couples intervention that is being developed in Pakistan to address fertility norms, mental health stress due to unpaid care work, and decision-making challenges with an objective of improving the quality of life of couples and family units. [34] The life skills intervention (PAnKH) implemented in Rajasthan, the site of our study, specifically aimed to empower adolescents, including newly married girls and young women. However, the intervention’s report highlighted formidable obstacles in engaging married girls, underscoring the imperative for innovative strategies to reach this hard-to-reach demographic. [35]

Previous research conducted in India has addressed the family planning and sexual reproductive health needs of married couples, yet neither program employed group-based interventions nor specifically targeted the specific needs of newly married women, as mentioned above. For instance, the CHARM2 program implemented in rural Maharashtra adopted a couple-focused intervention model, emphasizing women’s reproductive autonomy and delivering personalized care and counseling at the couple level, yielding improvements in reproductive and sexual health outcomes. [36] A program dating back over fifteen years, the First-time Parents Project, aimed to enhance the reproductive and sexual health knowledge, autonomy, and social support networks of a diverse group of young married women through integrated interventions during the early stages of marriage and pregnancy. Although positive results were observed in some indicators, findings on reproductive health outcomes were mixed.[37] Except for a handful of interventions [38–40], very few family planning interventions in the Indian context have targeted gender determinants such as deeply ingrained social norms surrounding fertility and gender roles, enhancing spousal communication, male involvement in family planning decisions, promoting women’s autonomy and empowerment over their reproductive health, and addressing concerns about contraceptive side effects.

In summary, the paucity of evidence on this neglected demographic and the persistent challenges faced by newly married girls and women, including early marriage and childbearing, restrictive gender norms, disempowerment, and poor family planning uptake highlight the pressing need to design and evaluate innovative interventions tailored to their specific context and requirements. Evidence suggests that life skills programs are most effective when customized to address the distinct needs of specific adolescent groups, involving their active engagement in the intervention process. [30] Therefore, our team aimed to develop a life skills and reproductive health empowerment intervention using a participatory approach with newly married girls and young women (18–25 years), rooted in their local and cultural context. Additionally, by involving key marital household stakeholders of newly married girls and women, such as husbands and mothers-in-law, we sought to develop an intervention that targets the structural and gendered determinants of sexual reproductive health, including women’s agency/autonomy, couples’ communication on family planning, and inequitable gender norms. Finally, by integrating life skills into a reproductive health empowerment intervention that goes beyond providing knowledge of sexual and reproductive health, we hypothesize that our intervention will empower participants to take control of their reproductive health, make informed decisions, and ultimately achieve their desired reproductive and family planning goals. This paper reports on the pilot findings of this intervention.

### 1.2 Study objectives

Feasibility studies are recommended to provide guidance in determining whether an intervention is ready for testing in a fully powered trial.[41] Hence, prior to launching the trial, we aimed to evaluate the feasibility and acceptability study of a life skills and reproductive health empowerment intervention, which will inform our larger well-powered rigorous trial. We described the development of the community-based participatory intervention in detail. Further, we used a comprehensive convergent mixed methods pilot study to evaluate the acceptability, feasibility, and challenges of implementing our intervention in villages in Rajasthan to inform the intervention, study design and operational details for a larger cluster-randomized controlled trial. [42] We also explored barriers and facilitators of intervention implementation from participants and program staff as well as the qualitative impact of the intervention on participants.

## 2 Materials and Methods

### 2.1 Development of the TARANG intervention for and with newly married women

**Figure 1:**
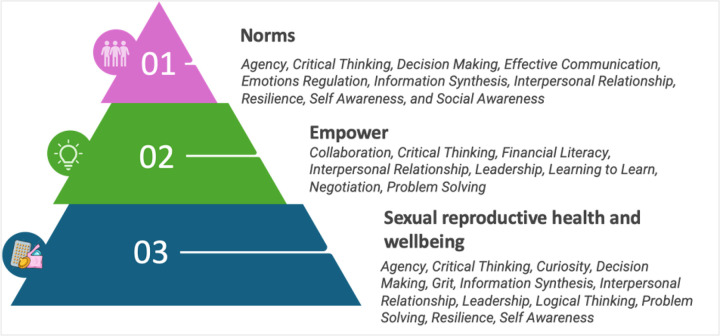
Life skills integrated into the TARANG intervention for newly married women

The TARANG intervention was developed and tailored to address the needs of young, newly married women in Rajasthan. The final curriculum for the intervention was rooted in the principles of adult and experiential learning with a rights-based framework, covering 19 specific life skills under three overarching thematic areas––Empower, Norms, and Sexual Reproductive Health and Wellbeing (Fig. 1), with more details in our protocol paper.[42] We prioritized these three thematic areas together in a series of consultations with our implementing non-governmental organization (NGO) partner, Vikalp Sansthan (referred to as NGO staff henceforth), based on their decades of field experience with newly married women to ensure it was directly applicable to young women’s lives. The curriculum, built on the rights-based framework, adopts a ‘spiral-curriculum approach’, wherein various aspects and topics are interconnected throughout the curriculum, serving to reinforce the message and deepen participants’ understanding of the interconnectedness among the key concepts outlined.

The curriculum development was led by the Orange Tree Foundation, an organization based in Jodhpur, Rajasthan, with contextual knowledge and extensive experience in working with young people and promoting youth-friendly educational programs. Given that this curriculum targets neo-literate participants residing in rural and tribal areas of Southern Rajasthan, the team from the Orange Tree Foundation meticulously designed the sessions to be interactive and activity-driven, with audiovisual aids and self-explanatory illustrated visuals. The content was developed directly in vernacular Hindi, tailored to the specific context of Southern Rajasthan, where our study is based, encompassing character names, illustrations, case studies, and cultural nuances pertinent to the region. To enhance the learning process, the TARANG toolkit, comprising of tools, posters, activity charts, link to audio-video resources and other relevant resource materials, was developed to serve as a resource compendium for moderators and participants. The toolkit also includes additional reading materials for the trainers that supplement in-session delivery.

During the developmental phase spanning from February to May 2023, we conducted iterative user testing with 38 newly married women in selected villages in Udaipur district of Southern Rajasthan, one of our primary study locations. Drawing insights from this pilot phase, final curriculum was developed by incorporating cultural adaptations and ensuring the curriculum’s linguistic suitability and appropriateness for the target audience.

### 2.2 Overview of the TARANG intervention

TARANG (Transforming Actions for Reaching and Nurturing Gender Equity and Empowerment) meaning, ‘cascading waves’, in Hindi, and was chosen because, through this curriculum, we hypothesized that young women would feel empowered through a ‘wave’ of agency to make informed choices about their bodies as well as family planning. The TARANG intervention was implemented by our NGO staff based in Udaipur, Rajasthan.

#### Moderators and groups

The Orange Tree Foundation team trained NGO staff and two female moderators in two phases, over a period of seven days, on the intervention curriculum in May and October 2023. Four intervention groups were run in four villages, with convenient timings for the groups set by each group individually. Moderators also continued to receive regular supervision from the NGO team.

The TARANG intervention started with an introductory rapport-building session, followed by 16 group sessions facilitated by the moderators over six months. Using practical skill-building activities, the intervention aimed to empower participants by enhancing their understanding of fundamental topics such as menstruation, conception, and contraception. Additionally, the curriculum was designed to strengthen participants’ sense of agency and their ability to make informed decisions regarding family planning and the timing of their first childbirth. Finally, the curriculum was designed to bring awareness to inequitable gender norms and their rights. The 16 sessions are summarized below:

The TARANG intervention also included separate, light-touch interventions for important household-level stakeholders, namely husbands and mothers-in-law, to create a supportive environment at home for the newly married women. The feasibility and acceptability of this part of the intervention for husbands and mothers-in-law will be published elsewhere.

### 2.3 Study design and setting

Mixed methods research designs integrate quantitative and qualitative data to harness the complementary strengths of both datasets and have a more comprehensive evaluation of intervention feasibility than would be attainable from either dataset alone. [43] This convergent mixed methods study consisted of a one group design with pre- and post-intervention data collection to examine the feasibility and acceptability of the intervention.

The study was conducted across four villages in Kumbhalgarh block of Rajsamand district of Southern Rajasthan in India from July 2023 through January 2024, with the intervention for women spanning six months. Kumbhalgarh block was chosen by the NGO partner based on their area of operations and cultural competency to work in these areas. While block level estimates are not available, Rajsamand district lags the state’s family planning indicators––only one in two women use any modern contraceptive method. [44] Further, in Rajsamand district, female sterilization is the dominant method adopted by women, which is a limiting family planning method, highlighting significant gaps in adoption of reversible short/long-acting contraceptive methods such as condoms, intrauterine devices, pills, among others. [44]

**Figure 2:**
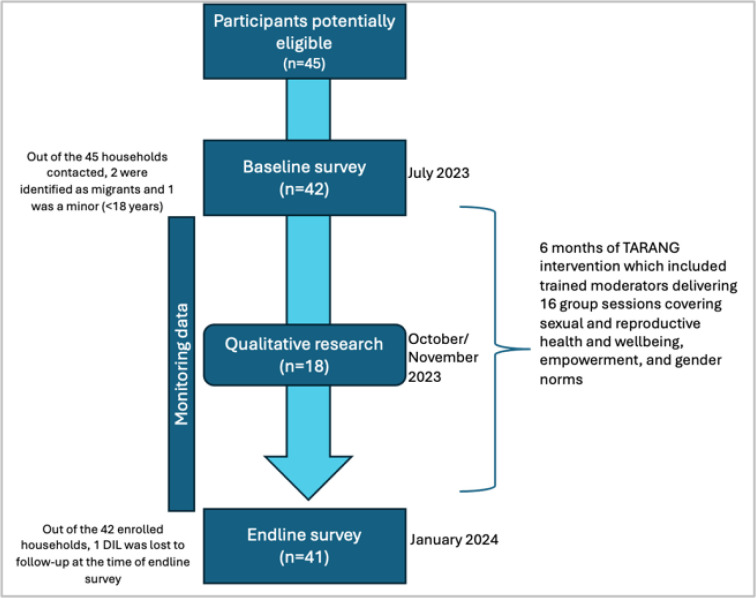
Timeline of TARANG intervention sessions and different modes of data collection

The sample size was based on feasibility given this was a pilot study. The eligibility criteria for the study included: married women between the ages of 18 and 25 who had been living in their husband’s home at least for the past six months, cohabiting with mother-in-law, and had been married within the last one year. Individual private consent was sought from newly married women. All participants who were approached agreed to participate but three participants did not meet eligibility criteria at baseline. A total of 45 potentially eligible households were approached and 42 newly married women (also called participants henceforth) were enrolled in the study at baseline (Fig. 2). Three households could not be enrolled because two were migrants and one participant was a minor (below 18 years of age). All participants provided audio-recorded verbal informed consent before all rounds of data collection.

### 2.4 Data collection

#### Quantitative surveys

We collected quantitative data through close-ended surveys with participants before the launch of the intervention (baseline) and after the end of the intervention (endline). Sex matched trained enumerators conducted surveys (~ 60–70 minutes) using structured computer-assisted personal interviews.

#### Qualitative data

From the study participants enrolled in the study, we purposively selected 12 participants for in-depth interviews (IDIs) and 6 NGO staff implementing and managing the intervention. We attempted to interview participants who had less than 25% attendance as well as those with greater than 50% attendance to understand a diversity of perspectives and experiences. In October 2023, at the middle of the intervention period, in-depth interviews were led by the first author (LG) in participant’s preferred language (Hindi), using an interview guide (see Additional file 1) in a private setting at the participants’ homes. We sought separate verbal consent from participants before conducting interviews. The average length of the interviews was ~ 35 minutes. Interviews were audio recorded and transcribed.

#### Monitoring data

Throughout the period of the intervention, we also collected monitoring data through a mobile application form that was completed after every session by moderators. The form captured the attendance and reasons for missing the intervention sessions.

The intervention timing and data collection are shown in Fig. 1 below.

##### Ethical approvals

Study protocols were reviewed and approved by institutional review boards at the University of California, San Francisco (IRB number: 22–37173), and the India-based Center for Media Studies (IRB00006230).

### 2.5 Quantitative measures

Quantitative data collected except for intervention perspectives at 6-months (post-intervention) were not reported in this manuscript due to our *a priori* emphasis on feasibility and acceptability as a primary study aim, including assessment and refinement of data collection tools and procedures for a future trial. Prior to initiating the intervention, we established *a priori* definitions for the primary outcomes, focusing on feasibility and acceptability to allow us to provide critical information for planning a larger randomized controlled trial.

1. Feasibility (proportion of participants who attended at least 50% intervention sessions)
2. Acceptability (proportion completely satisfied/somewhat satisfied with TARANG intervention)
3. Usefulness (proportion who found TARANG intervention useful/somewhat useful)

Retention rates were defined as women enrolled at baseline through the end of the study and are presented as percentages. Quantitative data were summarized as proportions using Stata 15.1. [45]

### 2.6 Qualitative themes and analysis

We analyzed in-depth interview transcripts line-by-line using a codebook. We developed a coding framework, deductively based on the interview guide, was iteratively refined with the addition of inductive codes following coding of initial transcripts. A team of three researchers initially double-coded at least 10% of the transcripts using the codebook in Dedoose version 9.0.107.[46] The first author (LG) queried and analyzed the code reports and developed themes along with illustrative quotes using a thematic analysis.[47]

### 2.7 Mixed methods integration

We employed a convergent mixed-methods approach, with the interview guide to complement and elucidate the quantitative results.[43] We present the quantitative and qualitative findings separately and integrate them using a joint display in the results section, followed by a synthesis of both in the discussion to derive insights. [48, 49]

## 3 Results

### 3.1 Participant characteristics at baseline

Forty-five participants assessed for eligibility and 42 participants were enrolled in the study at baseline, ranging in age from 18 to 24 years (mean 20.3, SD ± 1.6). Most (78.5%) had a marriage that was arranged by their parents. About 38% participants had received some primary schooling, over a third had secondary education and about 14% had received no education at all. Most participants belonged to the Scheduled Tribe (63.4%) or Scheduled Caste (14.6%).^[1]^ The majority (92.9%) wanted two or more children and about 71.4% wanted to wait at least two years before having their first child. About a quarter (~ 23.8%) reported ever used a method of contraception, highlighting the need for this intervention. Sociodemographic and fertility preference related characteristics are summarized in Table 2.

### 3.2 Quantitative results on feasibility and acceptability

Attendance to the groups was high––82% participants attended more than 50% of the sessions. Of those who attended at least one session, the median number of sessions attended was 14, i.e., 50% of the women attended at least 14 or more sessions (Interquartile range: 6–16), suggesting strong feasibility. Six of the 42 enrolled participants did not attend any session. In terms of acceptability, the majority (97%) of the participants were satisfied/somewhat satisfied with the intervention, highlighting overwhelming acceptability to the program. In terms of usefulness, all participants who attended at least one sessions found the TARANG intervention either to be useful or somewhat useful (Table 3).

A range of other intervention perspectives were sought using structured surveys as listed below in Table 4. Overall, there was a high level of satisfaction and positive perceptions across various aspects of the TARANG intervention. Forty one out of 42 participants recruited at baseline were retained through the study (97.6%). Out of those that attended at least one session, all (100%) expressed a high likelihood of recommending the intervention to their friends. All participants (100%) reported feeling a strong connection with other participants. All participants (100%) felt that the session duration was appropriate and did not want any changes. The majority (97.1%) of participants (34 out of 35) felt that the time between sessions were appropriate and the number of sessions was suitable. All participants (100%) believed that they could apply the information from the intervention in their daily lives. A significant number of (85.7%) participants reported feeling confident about making decisions regarding children and health. All participants (100%) reported feeling positive changes in themselves regarding decisions about having children and family planning. The majority (97.1%) of participants (34 out of 35) experienced a positive impact on communication and understanding with their husbands regarding family planning. All participants (100%) expressed liking the TARANG intervention.

### 3.3 Qualitative results

We present themes based on intervention acceptability and usefulness of participating in the intervention, and barriers and facilitators to implementing this intervention, and conclude with program improvements suggested by participants and NGO staff and moderators.

#### Theme 1: Acceptability of the intervention

Nearly all the participants reported that attending meetings made them feel “good”. The participants elaborated that meetings offered a sense of ease to sit and talk about family planning, menstruation, and other topics.

“Being a part of the program has provided me with new information that I didnt’ have before. I have been connected with new people, who I did not know earlier. I’ve gained new knowledge since joining.” – DIL 10, Age 22, Scheduled Caste

“It feels good. Everyone sits and talks….meetings should happen because we get information. These are topics we didn’t know before” – DIL 8, Age 19, Scheduled Tribe

Interviews with NGO staff and moderators underscored the evident necessity for an intervention such as TARANG for newly married young women, reflecting the program’s acceptance within the communities.

“There is definitely acceptance within people. The young married couples I talked with have found the need for such information. Young married couples dont’ have this kind of information. ….If they dont’ have this information and are able to grasp such information, then it is acceptable.” NGO staff 2

“People in my village share what they learn in meetings with each other, and it feels like they are learning something from us. Whatever we tell them, they are learning something new… Participants agree to continue with the program. I am receiving support from daughters-in-law, mothers-in-law, and even from husbands and fathers-in-law.” NGO moderator 1

Some women expressed appreciation for the TARANG intervention, as it equipped them with essential practical knowledge that had previously not been provided to them in any form, as well as increased mobility. They explained that given the gender norms in their villages, they were never allowed to step outside their homes to attend such sessions previously.

“I did not know before. Because we were not allowed to step outside the house. We used to go to school. Right after that, we used to go to the fields for work. They [parents] didn’t allow us to go anywhere.” DIL 10, Age 22, Scheduled Caste

#### Theme 2: Intervention’s usefulness

Many participants mentioned that the program was useful and was already bringing about tangible benefits in their life. The usefulness theme is described below in terms of their enhanced knowledge, decision-making and agency, new connections and social networks, relationship/communication with husband, and relationship with mother-in-law.

##### Usefulness: Knowledge

Overall, participants interviewed discussed benefitting from being part of the TARANG intervention, and none reported any negative consequences. Specifically, they noted that the curriculum was salient, increased their clarity on topics, and even introduced them to expanded knowledge previously not taught to them in schools or other settings.

“I enjoy attending the meetings; there’s no one else to inform us. I wasnt’ aware at first, but I learned from didi [moderator]. I’m following the advice.” – DIL 9, Age 18, Scheduled Tribe

Specifically, most participants discussed gaining awareness of contraception methods, including condoms, pills, injections, and Copper-T, highlighting their improved knowledge of different options available for family planning and contraception. They also discussed about gaining awareness of their overall health and other topics such as menstruation.

“We discussed about different contraceptive options—condoms, pills,… There should be a three-year gap between the birth of two children. When you don’t space children, the body gets weak.” DIL 12, Age 21, Scheduled Caste

“It has taught us that we need to be careful when such situations (pregnancy) arise with us.” DIL 6, Age 23, Scheduled Caste

Several participants expressed the importance of spacing between children, suggesting a preference for a gap of around three years between each child. This demonstrates an understanding of the benefits of spacing in terms of the health and well-being of both the mother and the child.

“There should be a gap of two to three years between children. It means the first one should grow up, and preparations for the second one should be made, taking care of oneself…It’s good that we discuss things related to our body parts; that’s correct.” DIL 3, Age 21, Other Backward Class

“It felt good when they talked about us, they told us about the problem of the womb, and used to look at pictures in science during studies. Eggs, and sperm are formed, and information is received.” DIL 6, Age 23, Scheduled Caste

Few participants also discussed the ideal number of children, with responses indicating a preference for having one or two children. This suggests that participants have more a thoughtful approach to family planning after attending intervention sessions, and now can consider factors such as maternal health, and the ability to provide adequate care and support to each child before planning their pregnancy.

Not only had participants acquired knowledge, but they had also learned to contemplate the consequences of engaging in unprotected sex. For example, one participant even mentioned feeling fearful of engaging in sex without contraception.

“Yes, I feel worried; there’s fear. Sometimes, engaging in relationships without contraceptives, child maybe conceived….” DIL 6, Age 23, Scheduled Caste

Interviews with the NGO staff also echoed TARANG intervention’s significant benefits, highlighting a common theme: prior to its implementation, newly married women lacked awareness of methods to prevent early pregnancies. The staff emphasized the necessity of interventions like ours, as these women had not previously been exposed to such awareness-raising initiatives.

Because what is the need to get pregnant at such a young age? The things they didnt’ know about, no one told them, whether it be about periods, having children, or contraceptive methods." NGO staff 1

Moderators who delivered the intervention also agreed that women had benefitted immensely from attending the TARANG intervention sessions. Specifically, they noted the benefit of activities done to teach the sessions to young newly married women. The “seed activity” conducted during one the TARANG sessions, which demonstrated how the gender of a child is determined, proved to be a meaningful and impactful experience for the participants. Through this activity, participants gained insight into the biological processes that influence the sex of a child, leading to a deeper understanding of the natural factors at play.

“When we conducted sessions, they didnt’ know before how pregnancy occurs, what a boy is like, and what a girl is like. Through activities, they are getting this kind of information. Through activities like “seeds”, they understand that there are two types of seeds, one for men and one for women. They now understand menstruation, are aware of their health, and know the benefit of spacing in children, including family planning awareness”. NGO moderator 1

##### Usefulness: Decision-making process and sense of agency

Few participants expressed a sense of newfound autonomy and agency in decision-making, particularly regarding matters related to reproductive health and family planning. They highlighted the shift from seeking approval or guidance from family members, such as mothers-in-law and husbands, to making independent decisions based on their own preferences and desires. The intervention encouraged participants to engage in open dialogue with their spouses, families, and communities, fostering a collaborative approach to decision-making. The participants felt that they could assert their own preferences and make decisions that aligned with their individual needs and goals. They reported a sense of empowerment to make more informed decisions about family planning and contraception.

“Earlier, I couldnt’ make decisions on my own. I used to ask my mother-in-law and husband. If it’s about both of us, we make decisions by asking each other. If it is about my mother-in-law, I will ask her. If it is about anyone else in the family, I will consult with all the family members. And if it concerns me, I can make my own decision.” DIL 5, Age 25, Scheduled Caste

A few participants emphasized the importance of husband and wife jointly making decisions, indicating a recognition of the equal partnership and shared responsibility in decision-making within marital relationships.

Prior to the intervention, their approach to family planning may have been more passive or influenced by traditional norms. However, after gaining knowledge and critical thinking skills through TARANG sessions, they have become more proactive and deliberate in their approach to family planning. One participant mentioned considering factors such as the timing of childbirth and the desired age gap between children, suggesting a more thoughtful and informed decision-making process, as discussed below:

“There has been an impact on our family planning perspective, Earlier, we used to think about something, but now we think differently - When should we have a child, how much age gap should be there between children, etc.” DIL 3, Age 21, Other Backward Class

##### Usefulness: New connections and establishment of social networks

Many participants experienced discussed benefits from interacting with one another, forging connections, and establishing social networks within the community. Through these interactions, participants reflected on their ability to share experiences, exchange information, and provide support to one another, leading to a sense of camaraderie and belonging.

“We make friends by attending the meeting. We learn how to talk, get to know each other, and understand our bodies. We didnt’ know moderator madam before, but after joining this meeting, we got to know each other…All my friends are those who attend the meeting. As we get to know each other, we can laugh and joke together, and talk with each other.” DIL 3, Age 21, Other Backward Class

##### Usefulness: Relationship with husbands/ couples’ communication

A few participants reported that they shared information discussed during TARANG sessions with their husbands. This communication facilitated a deeper understanding and mutual support regarding topics related to reproductive health and family planning within their marital relationships.

“Yes, it is significant. I mean, if we have to make an important decision or if we are not liking something, we should speak openly. Likes and dislikes should be communicated. One should communicate their problems to their husband, to friends, especially to the husband… Like, we both take each other’s opinions. We work by consulting each other. It was a bit less at first, but now it’s fine” DIL 3, Age 21, Other Backward Class

##### Usefulness: Relationship with mothers-in-law

In addition to the noted benefits in their relationships with their husbands, some participants highlighted improvements in their relationships with their mothers-in-law. It is possible that the TARANG intervention’s dedicated sessions involving mothers-in-law are also contributing to fostering understanding and harmony between daughters-in-law and mothers-in-law. This suggests a positive impact on family dynamics, promoting communication and support within the household.

“Like, when we are going to the meeting, my mother-in-law and husband listen to me, and I also listen to them. Everything is going well….In the session, we learned that one should consider their daughter-in-law as a daughter. For example, our mother-in-law considers her daughter, who got married, as a daughter-in-law, just like they (mothers-in-law) should consider us daughter-in-law as their daughter, and that’s how we receive love.” DIL 3, Age 21, Other Backward Class

#### Theme 3: Barriers and facilitators (participant perspectives)

Participants noted a few barriers to attending sessions such as busy routine lives, time, and availability to come to sessions.

##### Workload, daily routines, and household responsibilities

Most participants’ lives revolve around household chores and agricultural farm labor work or cattle rearing with very little rest time or leisure time. Often, this busy schedule made it challenging for the participant to attend sessions regularly.

“I make roti [bread], fetch fodder for the buffalo, and eat roti [bread]. Bringing fodder for the goat, then taking a two-minute rest, going to the farm at three-four o’clock, and in the evening, making roti again” DIL 9, Age 18, Scheduled Tribe

A few participants faced additional care responsibilities, such as caring for sick father-in-law or assisting their mother-in-law with household tasks, which further restricted their ability to attend sessions.

“He is ill at home, plus the guests come. We attend them. My mother-in-law will come after Navratri [festival]. Added to this, recently, my father also underwent surgery due to a lump in his urinary tract. So, I have to take care of both the houses [paternal + in-laws]. I go to my father’s place at night and return [to the in-laws] in the morning. There is grass-cutting work too, but I don’t do that. I primarily take care of the work at home.” DIL 5, Age 25, Scheduled Caste

##### Some resistance from husbands/fathers-in-law

While most participants felt supported to attend the sessions, a couple of participants said that they faced resistance from their husbands or fathers-in-law in attending sessions. In one instance, a participant’s husband discouraged her from attending, preferring she focus on household chores.

“My husband says that they teach you different things and they teach me different things.. He says that I should do household chores: rather than sit in the meetings.” DIL 2, Age 22, Scheduled Tribe

Moderators echoed that sometimes family members objected to women participating: “If we go to call these women every day, their families, particularly fathers-in-law, object, citing health concerns.” NGO moderator 2

A few participants navigated conflicting priorities and obtaining permission to attend the sessions. Despite facing occasional household chores or responsibilities, one participant expressed their desire to attend the sessions, indicating the importance she places on participating in the program, as noted below:

“They usually say to go, sometimes there is work, they ask me to come a bit early…They do give permission; even if they dont’ say, I still go. Sometimes I go without asking. Madam [moderator] comes, and if she asks, they say, "Go, come on time." DIL 6, Age 23, Scheduled Caste

##### Distance to the meeting venue for participants

A few participants mentioned that some women in their group lived away from the meeting venue which often makes it hard for them to attend.

“It takes 15 minutes for women in my group to arrive at the meeting because they live far away.” DIL 12, Age 21, Scheduled Caste

##### Strong desire to attend meetings to navigate barriers

Despite these challenges, a few participants expressed a strong desire to attend sessions, saying that they managed their time by working around their schedules to attend these meetings, highlighting the value these sessions were generating for them.

“No, still, we manage time for the meeting. There is no big deal with work, as Work is never-ending.. Whenever there was time, we used to ask about the meeting time beforehand. We allocate two hours for the meeting. If there is any work, we complete it before attending.” DIL 3, Age 21, Other Backward Class

#### Theme 4: Barriers and facilitators (NGO staff and moderator perspectives)

##### Challenges in recruiting newly married women for the study

NGO staff and moderators explained the concept of “gauna or aana” in Rajasthan, wherein newly married brides transition to their in-laws’ home and consummate their marriage, typically after a period, varying from several months to perhaps a year or more, rather than immediately after marriage. This made it challenging for the team to find “eligible” households for the study. Despite this challenge, we were on average able to recruit about 10–11 women in our sample for the pilot study.

“In our area, there’s a custom where newlywed brides move to their husband’s family home, called ‘gauna’ or ‘aana’. This usually happens after some time, ranging from a few months to even a year after marriage. Different communities have their own timings for this based on regional traditions. It’s quite common for brides to stay with their parents for a while before shifting to their husband’s place. This made it tough for us to find suitable households for our study. We needed married women aged 18 to 25 who had been living with their husband for at least six months and living with their mother-in-law. But because of the varying timing of ‘aana’, it was tricky to find households that met all our criteria. Plus, we also had to consider that the marriages had to be within the past year, which added another layer of complexity." NGO Staff 2

##### Limited time availability for newly married women to engage with the intervention

Moderators also echoed that participants had limited time to attend sessions due to their demanding daily routines and chores. They mentioned that many prioritize household tasks over attending meetings, fearing that neglecting these responsibilities will lead to household work suffering due to lack of time. Moderators also shared that because a significant portion of participants are engaged in labor-intensive work, it is difficult for them to attend meetings. Finally, they stressed that the ongoing grass-cutting season at the time of the qualitative study (it is seasonal and intensive labor work in Rajasthan often undertaken by women) added to the participants’ workload and time constraints.

“Today, these same women say, we have learned a lot. But it’s quite challenging to call women for meetings as they are busy with fieldwork. Sometimes when I invite them to a meeting, they respond, we must go home to our guests. The biggest challenge is that 90 per cent give more priority to household chores, fearing that if they attend the meeting, the household work will suffer, due to a lack of time.” NGO moderator 1

“For example, if the daughter-in-law comes home, and if the mother-in-law has small children, then if the mother-in-law goes somewhere, the daughter-in-law has to take care of them. Currently, the grass-cutting season is going on, so they are busy with that too.” NGO moderator 2

##### Initial shyness and need for rapport building

Moderators mentioned how shyness among newly married women posed a barrier at first. They talked about participants being hesitant to speak up during group sessions or ask questions due to feelings of embarrassment or shyness. This inhibits their ability to fully participate and benefit from the sessions.

“At first, they hesitate to speak, and if they dont’ understand, they dont’ ask again because they feel embarrassed” NGO moderator 1

In a few cases, moderators took the effort to engage participants by asking the women’s mothers-in-law to encourage them and support the newly married women in overcoming their shyness. This also potentially reassured women to engage in the intervention because they felt they had permission of the mothers-in-law.

“Initially, newlywed brides wouldnt’ talk to us, and wouldnt’ even disclose their names. When asked something, they would become shy and wouldnt’ share anything. Even in the sessions, there was hesitation. Even after repeated questioning, they wouldnt’ respond. Due to shyness, they would hide in someone else’s house. Then, we spoke to their mother-in-law to encourage them (women/their daughter-in-law) to talk.” NGO moderator 2

##### Festivals, weddings, events

Moderators highlighted how religious functions and festivals pose significant obstacles to conducting sessions effectively. There are certain months such as Sawan (monsoon), festivals (such as Holi, Diwali, etc.) when women are particularly engaged in religious and cultural activities. During these events, women participate in village gatherings, fairs, and religious rituals, which takes precedence over attending sessions. Often local fairs and community events involve extended periods of absence from their husband’s home, further making it challenging to schedule and conduct sessions effectively.

“The biggest challenge is that we are including DIL members whose marriage happened just six months ago. In this case, she will be busy with her piyar [parent’s home] for a month during the first Sawan. If she is associated with the Gameti community, there is a Gavari program for her, and Navratri (another Indian festival celebrated for 9-days in the month of Oct/November) is also there. To highlight a significant obstacle, attending DIL sessions for the entire month of Holi is challenging.” NGO Staff 1

##### Newly married women’s extended period of stay at their natal homes

The transitional period for newly married brides, as they acclimate to their marital homes, often results in extended stays at their natal/parental homes, presenting a significant barrier to their consistent attendance at sessions.

“Daughter-in-law (DIL) visits their parents quite frequently, due to being newly married, she stays in the in-laws’ house for some time and sometimes in her parental home.” NGO Staff 2

In some cases moderators facilitated women’s attendance by going and personally escorting them.

“Many women dont’ come on time; we have to go and call them, sometimes even escort them back” NGO moderator 1

“Our colleagues bring them sitting on a scooter, many times when the husbands do not have the resources, both the husband and wife are brought for the session.” NGO moderator 3

#### Theme 5: Suggestions for intervention improvement

A few intervention improvement themes emerged from interviews with both participants and NGO staff.

##### Themes in the meetings/sessions

Most participants agreed that the TARANG intervention was catering to their desire to learn and already covering topics of their interest.

The moderators also felt the TARANG intervention was appropriate, and the content was just “right”, although they highlighted that the intervention should be sensitively delivered.

“The content is right. It is essential how sensitively it is being taken to them. There are some topics where it is ensured that the trainer’s bias doesn’t come, and I do not give a wrong message. The content is based on the subject we are talking about.” NGO Staff 1

##### Length/time of the meetings

A few participants felt that the meeting length could be increased: “It should be increased. The more time we sit for the meeting, the more we will talk.” DIL 9, Age 18, Scheduled Tribe

Another woman described liking having ample time: “We discuss so much. It feels good when moderator madam says something, we can sit together, and talk” DIL 10, Age 22, Scheduled Caste

NGO staff also felt longer duration of sessions would be beneficial as it would allow them to listen to more of what women have to say.

“If the session duration is increased.. During the session, participants also share personal experiences, so it’s important to listen to them too” NGO moderator 2

##### Conduct of sessions by moderators

Nearly all participants expressed appreciation for the moderators’ effectiveness in delivering the sessions. They commended the moderators for their ability to explain topics in a language that participants could understand and for contextualizing the information to make it relevant to their lives. They were satisfied, stating that the moderators maintained a calm demeanor. The participants reported that they enjoyed the engaging nature of the sessions.

“Madam’s way of explaining is correct; she explains to us in our language. She keeps the sessions very lively - fun and not serious. I like her teaching very much.” DIL 5, Age 25, Scheduled Caste

“Yes, it’s good, absolutely perfect. She explains things calmly; for example, if we didnt’ understand something in the meeting yesterday, she explained it again. When she asks us questions, it’s good to answer.” DIL 3, 21 years, Other Backward Class

##### Intervention delivery to include videos

When probed about the benefit of videos, most participants felt that videos would enhance their understanding of the topics. They likened videos to stories, indicating they found them engaging and effective for learning: *“.. We can watch; we can watch at night. Watching videos will help in understanding, it’s like a story only.” DIL 6, Age 23, Scheduled Caste*

Specifically, one participant appreciated the use of various multimedia methods such as pictures, stories, case studies, and audio during meetings, suggesting a preference for diverse instructional approaches: *“In the meeting, things are explained through pictures, stories, case studies, and audio and all these methods are fine”. DIL 7, Age 19, Other Backward Class*

In terms of the intervention delivery, NGO staff and moderators also echoed the benefits of the videos, as they perceived it resonated better with women.

“There should be more videos because the one who listens may not be understanding it well.” NGO moderator 2

“After showing them the videos, we can discuss them with them. This way, their interest will be maintained in watching the videos, whose language is easy, and understandable”. NGO Staff 1

### 3.4 Data integration

Following the mixed methods methodology outlined by Guetterman, Fetters, and Creswell, we utilized a side-by-side joint display to compare qualitative and quantitative data and draw inferences regarding confirmation or discordance between the two types of data. [50] In Table 5, for each of the primary outcomes, we created the display using exemplar quotes from participants and staff interviews and reported the corresponding quantitative variables.

## 4 Discussion

To our knowledge, this is the first mixed methods study to assess the feasibility of a life skills and reproductive health empowerment intervention among newly married women in rural communities in low-and-middle-income countries. A community-based participatory intervention was developed with inputs from newly married women and implemented in rural/tribal Rajasthan. Through the mixed methods approach, we present a comprehensive understanding of the feasibility, acceptability, and perceived benefits of the TARANG intervention. Our quantitative and qualitative findings were aligned to suggest that the 16-week intervention over a six-month period was acceptable, feasible, and useful to participants.

The quantitative results from our feasibility and acceptability assessment indicate strong support for the TARANG intervention among participants, with over 80% of participants attending half of the sessions and overwhelmingly high satisfaction. Additionally, all participants found the intervention useful, highlighting the intervention’s practical value. Other feasibility outcomes assessed further underscored the positive reception of the intervention, with high retention rates, willingness to recommend the program to others, and positive feedback on session duration and content. We found that the program’s acceptance within the communities was underscored by the endorsement of NGO staff, who highlighted the evident necessity for such interventions among newly married women. This is especially crucial since past studies have had challenges in recruiting and retaining newly married adolescent girls and women in similar populations. [35] While we don’t have comparable studies from India or even South Asia, our results are especially promising given that no intervention to date from India has specifically targeted the sexual reproductive health needs or skill building of newly married adolescents girls and women.

Complementing these quantitative findings, the qualitative analysis provided insights into the participants’ experiences and perceptions of the TARANG intervention. The themes shed light on the acceptability and benefits perceived by participants. The overwhelming response from qualitative study was positive, with participants expressing appreciation for the opportunity to engage in open discussions about sensitive topics such as family planning and menstruation, and a sense of community and support. The intervention was perceived as filling a crucial gap in knowledge, providing practical information that was previously unavailable to participants. The qualitative findings also elucidated the tangible benefits experienced by participants because of their engagement with the intervention. Participants reported gaining new knowledge and awareness about contraception methods, reproductive health, and decision-making autonomy. The intervention facilitated not only individual learning but also the establishment of social networks, fostered interpersonal connections, and improved communication within marital and familial relationships. These findings highlight the holistic impact of the TARANG intervention on participants’ lives, beyond knowledge.

Although the findings of this study suggest that the intervention was positively received, our qualitative analysis revealed several barriers to consistent participation. NGO staff/moderators encountered challenges, including recruiting sufficient newly married women for the intervention, delivery-related challenges due to women’s busy daily routines, logistical constraints, cultural norms dictating extended stays at natal households, festivals/events, limited time availability due to household responsibilities, and the need for longer rapport building. Similar findings have been found in other studies involving women’s groups. [26] These barriers prompted us to refine the intervention for the main trial, incorporating flexibility to improve participant engagement.

The combined feedback from participants and NGO staff and moderators, as well as the deeper insights gained from integration of qualitative and quantitative data have informed changes to the intervention to the main trial, mainly to improve participant attendance and retention. Based on their feedback, we reduced the number of sessions from 16 to 14, including increasing the rapport building sessions (from 1 to 2). This was mainly done from NGO staff feedback to ensure participants had more time to engage with each other and feel more comfortable discussing sensitive topics with the moderators and amongst each other. In the first session, we also decided to introduce participants to their local public health system functionaries such as Community Health Workers and Nurse-Midwife in their marital villages to ensure they know how to access family planning services locally. We also included flexibility by allowing the sessions to not follow a weekly or bi-weekly pattern to accommodate women’s busy life schedules––the NGO staff will deliver at least two intervention sessions in a month to ensure completion of the intervention within 5–6 month time frame. The new schedule of sessions with a focus on understanding pregnancy as a choice, family planning and women’s agency (see Attached File 2). Since both participants and NGO staff expressed enthusiasm for videos and recognized benefits of including videos to facilitate comprehension, we included educational, and behavior change communication videos for every session. Further, all sessions have been made more engaging through use of various methods, including pictures, stories, case studies, and audio, in explaining concepts during sessions. While most participants found session length adequate, there was a desire among some from qualitative data for longer meetings to facilitate deeper discussions. Hence, we increased the duration of each session to now have an average of 75 minutes.

The main limitation of the study is that we did not have pre-set benchmarks as to what rates of participation and attendance we would use to deem the pilot intervention feasible because we did not have a similar study from India with which to compare, but this can be addressed in future research. By design, the sample size of this study was limited as the main purpose was to test the feasibility. We assumed, *a priori*, that a sample size of 45 participants would be enough to test all procedures of the study protocol. Overall, this number of participants proved to be sufficient in terms of testing the procedures and estimating rates of attendance and retention. However, we only tested in one district of India among women in 4 villages, findings may be different in other cultural contexts. Finally, we were also limited in our ability to report recruitment rates because a proper listing of eligible households was not carried out, however, qualitatively we were able to gauge the challenge of recruiting this elusive population.

Despite these limitations, our study provides valuable insights for the design and implementation of future interventions aimed at improving reproductive health outcomes in rural contexts and offers several strengths. First, the study is a comprehensive mixed methods approach, combining quantitative surveys, in-depth interviews, and monitoring data to fully grasp the feasibility and acceptability of the TARANG intervention. Second, our intervention development was informed by adult-learning principles and guided by iterative user-feedback from the community, ensuring a participatory approach throughout. This dual emphasis on community-based design and rigorous pilot research adds strength to our findings. By sharing our detailed intervention development process, we aim to contribute to the broader field of implementation science, enabling researchers and practitioners to replicate and adapt similar interventions in diverse settings. Third, by assessing feasibility and acceptability in a select yet representative group of villages in rural/tribal Southern Rajasthan, where the larger trial is slated to occur, our study provides valuable insights for the forthcoming larger-scale randomized controlled trial.

### Conclusion

The TARANG intervention, a life skills and reproductive health empowerment intervention, developed through a community-based participatory process with inputs from newly married girls and young women, demonstrated high acceptability and feasibility among newly married women in rural/tribal communities in Rajasthan. Our mixed methods approach, integrating quantitative surveys, monitoring data, and qualitative interviews, revealed strong support for the intervention, with participants reporting high levels of satisfaction, perceiving it as valuable. The intervention also made several positive impacts on participants’ lives in terms of increasing knowledge on reproductive health and family planning, communication, social connections, and empowerment. We constructively used the challenges to consistent participation to inform refinements to the intervention design for the forthcoming main trial, including adjustments to session frequency, content, and duration. The integration of participant and NGO staff/moderators feedback, alongside the combination of qualitative and quantitative data, improved our understanding and informed modifications to enhance the intervention’s effectiveness and reach for the larger trial.

## Figures and Tables

**Table 1:** TARANG intervention sessions and objectives

Session #	Name of the session	Objectives
Session 0	Kickstart	Building rapport, fostering a culture of learning
Session 1	My health, my priority	Prioritizing health and understanding its four pillars: mind, body, heart, and soul
Session 2	Know your body	Understanding menstruation, menstrual health management and conception
Session 3	Science behind conception	Engaging in activities to comprehend sex determination of the fetus and addressing norms related to son preference
Session 4	Contraception	Exploring various contraceptive methods tailored to individual needs and life circumstances.
Session 5	Love has no space for violence	Developing a support system chart and recognizing different forms of violence, including physical, sexual, economic, and emotional
Session 6	My identity	Reflecting on personal identity and self-awareness
Session 7	Responsible sexual behavior	Addressing issues such as hygiene, sexually transmitted infections, and reproductive tract illnesses
Session 8	My consent is important	Exploring the concept of consent, emphasizing voluntary and informed decision-making, and understanding the right to withdraw consent
Session 9	How to choose family planning methods	Understanding that family planning is a shared responsibility and should be a personal decision
Session 10	Negotiate your way	Examining healthy and unhealthy relationship dynamics and developing negotiation skills
Session 11	Language of money	Learning to create a family budget and strategies for saving money
Session 12	Preparing for a digital world	Understanding essential digital tools such as Gmail, phone apps, and utilizing search engines effectively
Session 13	My five year plan	Engaging in a workshop to make decisions for the next five years, including considerations for family planning
Session 14	Conception and abortion	Exploring topics related to abortion, sex-selective abortion, and relevant laws
Session 15	Safe maternity, healthy life	Understanding conception, pregnancy, antenatal care, and newborn care
Session 16	Managing emotions and stress	Learning coping mechanisms to manage stress effectively

**Table 2:** Baseline demographic characteristics and fertility preferences (n = 42)

Demographics	% / mean (SD)	n
Type of marriage		
Arranged	78.5	33
Love	21.4	9
Age	20.3 (± 1.6)	42
Education		
No education	14.3	6
Primary education (1–8)	38.1	16
Secondary education (9–12)	33.3	14
Higher education (any college)	14.3	6
Caste		
Schedule caste (SC)	14.6	6
Schedule tribe (ST)	63.4	26
Other backward class (OBC)	12.2	5
General category (GC)	7.3	3
Work		
Never gainfully employed	59.5	25
Gainfully employed but not currently	35.7	15
Currently gainfully employed	4.8	2
Number of children participant wants		
No preference	7.1	3
2 or more	92.9	39
Ideal time to childbirth		
No preference	11.9	5
< 1 year	16.7	7
2 or more years	71.4	30
Want to wait at least 2 years for first child	71.4	30
Son preference		
More boys	54.7	23
More girls	16.7	7
No preference	28.6	12
Ever used contraception	23.8	10

**Table 3:** Primary outcomes of TARANG intervention

Primary outcomes		%	n
Feasibility	Proportion of participants who attended at least 50% of the sessions	82.0%	41
Acceptability*	Proportion completely satisfied/somewhat satisfied with TARANG intervention	97.1%	34
Usefulness*	Proportion who found TARANG intervention useful/somewhat useful	100.0%	35

* Calculated based on 35 participants who attended at least one session.

**Table 4:** Other outcomes assessed for the pilot intervention (n = 42)

Other outcomes assessed (%)	%	n
Retention rate^#^ (%)	97.6%	41
Very likely recommend a friend to join the TARANG intervention	100.0%	35
High level of connection with the other participants during the TARANG intervention sessions	100.0%	35
Perceived the session was just right	100.0%	35
Perceived interval between two sessions in the TARANG intervention was just right	97.1%	34
Perceived right number of sessions held in the TARANG intervention	97.1%	34
Perceived information from TARANG intervention could be applied in their daily life to great extent/some extent	100.0%	35
Perceived confidence about making decisions about timing of children and health after participating	85.7%	30
Perceived positive changes in themselves regarding making decisions about having children and family planning after participating in the TARANG intervention	100%	35
Perceived positive impact of TARANG intervention on communication and understanding between participant and their husband regarding family planning	97.1%	34
Liked the TARANG intervention	100%	35
Want sessions of TARANG intervention to be held in their village	100%	35

# Of the 42 participants enrolled at baseline, 1 participant was lost-to-follow-up at endline due to migration.

**Table 5:** Joint Display Table for Integration of Quantitative and Qualitative Findings

Implementation outcomes	Qualitative results and exemplar quotes from participants	NGO/program staff interview results	Quantitative results	Mixed methods interpretation
Feasibility	Most participants expressed feeling positive about attending meetings, but also some noted busy daily routines, distance to the meeting venue for participants among others. A few instances of resistance from husbands/fathers-in-law were noted. Yet participants also had an extremely strong desire to make it to meetings. *“No, still, we manage time for the meeting. There is no big deal with work, as Work is never-ending. Whenever there was time, we used to ask about the meeting time beforehand. We allocate two hours for the meeting. If there is any work, we complete it before attending.” DIL 3, 21 years, Other Backward Class*	NGO staff and moderators felt the program was feasible to do, though mentioned barriers in conducting intervention sessions including, festivals/events, limited time available with newly married women/burden of household responsibilities, women’s stay at their natal households, shyness and need for rapport building. *“Participants agree to continue with the program. I am receiving support from DIL, MIL, and even from husband and father-in-law” NGO staff 1*	• 82% participants attended more than 50% of the sessions. • 100% participants liked the TARANG intervention. • 100% participants wanted sessions of TARANG intervention to be held in their village.	Despite the noted barriers, we observed an overwhelmingly strong alignment between quantitative findings and qualitative reports from participants and staff.
Acceptability	Almost all participants were appreciative of the TARANG intervention, citing a sense of ease in discussing topics like family planning and menstruation, which was previously never available to them. Further, they shared information learned in meetings with others. *“Being a part of the program has provided me with new information that I didn’t have before. I have been connected with new people, who I did not know earlier. I’ve gained new knowledge since joining.” - DIL 10, Age 22, Scheduled Caste*	Interviews with NGO staff and moderators underscored the necessity of TARANG among newly married young women, indicating community acceptance. Staff noted the lack of information among young couples and the significance of the program in filling this gap. *“People in my village share what they learn in meetings with each other, and it feels like they are learning something from us. Whatever we tell them, they are learning something new...* *Participants agree to continue with the program..” NGO moderator 1*	• 97% participants completely satisfied/somewhat satisfied with TARANG intervention. • 100% participants will very likely recommend a friend to join the TARANG intervention.	Confirmation of alignment between a priori indicator in quantitative findings and qualitative reports from participants and staff.
Usefulness of the intervention defined as proportion of participants who found the TARANG intervention sessions useful or somewhat useful	Intervention was useful to participants in improving their knowledge of family planning, developing a sense of autonomy and decision-making, even joint- decision-making about, building new connections, sense of agency, and better communication/relationship with husbands and mothers-in-law. **Connections** “We make friends by attending the meeting. We learn how to talk, get to know each other, and understand our bodies. We didn’t know moderator madam before, but after joining this meeting, we got to know each other...All my friends are those who attend the meeting. As we get to know each other, we can laugh and joke together, and talk with each other.” DIL 3, Age 21, Other Backward Class **Knowledge** *“There should be a gap of two to three years between children. It means the first one should grow up, and preparations for the second one should be made, taking care of oneself...It’s good that we discuss things related to our body parts; that’s correct.” DIL 3, Age 21, Other Backward Class* **Decision-making/ Agency** *“Earlier, I couldn’t make decisions on my own. I used to ask my mother-in-law and husband. If it’s about both of us, we make decisions by asking each other. If it is about my mother-in-law, I will ask her. if it is about anyone else in the family, I will consult with all the family members. And if it concerns me, I can make my own decision.” DIL 05, Age 25, Scheduled Caste*	NGO staff and moderators highlighted the impact of the TARANG intervention, noting that prior to its implementation, newly married women lacked awareness of pregnancy as a choice and contraceptive methods. They stressed the importance of such initiatives, emphasizing the need to address gaps in knowledge concerning menstruation, childbirth, and contraception. *“They are becoming aware of health. When we conducted sessions, they didn’t know before how pregnancy occurs, how a boy is born, and how a girl is born. Through activities, they are getting this kind of information. Through activities like seeds, they understand that there are two types of seeds, one for men and one for women. They now understand menstruation, are aware of their health, and know the benefit of spacing in children, including family planning awareness.” NGO moderator 1*	• 100% perceived the TARANG intervention sessions to be useful or somewhat useful. • 85.7% participants felt confident about making decisions about timing of children and health after participating. • 100% participants perceived a high level of connection with the other participants during the TARANG intervention sessions. • 100% participants felt positive changes in themselves regarding making decisions about having children and family planning after participating in the TARANG intervention. • 97.1% participants felt very positive/little positive impact of TARANG intervention on communication and understanding between participant and their husband regarding family planning. • 100% participants felt information from TARANG intervention could be applied in their daily life to great extent/some extent.	Confirmation of alignment between outcomes in quantitative findings and qualitative reports from participants and staff.
Improvement to the program	Participants expressed satisfaction with the TARANG intervention, noting that the topics covered aligned with their interests and were appreciative of the moderators’ efforts at creating engaging sessions. Overall, they expressed enthusiasm for incorporating videos into the intervention delivery, as they believed it would increase comprehension. *“.. We can watch; we can watch at night. Watching videos will help in understanding, it’s like a story only.” DIL 6, Age 23, Scheduled Caste*	Moderators also felt the intervention’s content was appropriate, but also noted some improvements such as, increasing the duration of the sessions, incorporating videos, and emphasized more rapport building sessions. *“if the session duration is increased.. During the session, participants also share personal experiences, so it’s important to listen to them too” NGO moderator 2*	•100% who felt the session was just right. • 97.1% who felt interval between two sessions in the TARANG intervention was just right. • 97.1% who felt right number of sessions held in the TARANG intervention.	There were some discrepancies between the quantitative results and some of the qualitative data in terms of program improvements.

## Data Availability

The quantitative data that support the findings of this study are available on request from the corresponding author. The qualitative data, given the small sample size, is not available to protect the identity of participants.

## Key messages regarding feasibility

- Newly married girls and young women face many challenges but there is limited evidence on interventions that work to help them delay childbearing and increase uptake of family planning. A life skills education-based reproductive health empowerment intervention was developed and delivered to newly married women in a group format.
- TARANG intervention, developed using a community-engaged approach, was a feasible and acceptable among this population.
- The findings imply that similar interventions could be adapted and tested in similar contexts to address reproductive health needs and empower newly married women. However, flexibility is required in addressing barriers to implementation, such as women’s workload, and cultural norms of being in natal homes for extended periods among others, and intense rapport building to optimize women’s participation and effectiveness.

## Footnotes

1. For caste, following the Government of India classifications, we asked participants to report their caste. We categorized it as scheduled tribe, scheduled caste, other backward class (often grouped as marginalized castes) and general caste.
